# An Autologous Human Adipose Stem Cell-Derived 3D Osteogenic Implant for Bone Grafting: From Development to First-in-Human Experience

**DOI:** 10.3390/jcm14186436

**Published:** 2025-09-12

**Authors:** Torsten Gerich, Pierre-Louis Docquier, John A. Carrino, Mikael Boesen, Nadine Schmid, Ginny Hsu, Ji-Hye Yea, Aaron James, Judy Ashworth, Hara Episkopou, Denis Dufrane

**Affiliations:** 1 Centre Hospitalier de Luxembourg, 4 Rue Ernest Barblé, L-1210 Luxembourg, Luxembourg; gerich.torsten@chl.lu (T.G.); sec.chirplas@chl.lu (N.S.); 2 Department of Orthopedic and Trauma Surgery, Cliniques Universitaires Saint-Luc, Université Catholique de Louvain—UCLouvain, 1200 Brussels, Belgium; pldocquier@gmail.com; 3 Weill Cornell Medicine, Hospital for Special Surgery, New York, NY 10021, USA; carrinoj@hss.edu; 4 Department of Radiology, Frederiksberg Hospital, Nordrefasanvej 57, 2000 Frederiksberg, Denmark; mikael.boesen@gmail.com; 5 Pathology Department, Johns Hopkins University, Baltimore, MD 21218, USA; hsug@ohsu.edu (G.H.); jyea2@jh.edu (J.-H.Y.); awjames@jhmi.edu (A.J.); 6 Novadip Biosciences, 1435 Mont-Saint-Guibert, Belgium; judy.ashworth@novadip.com (J.A.); denis.dufrane@novadip.com (D.D.)

**Keywords:** autograft, bone remodeling, bone substitutes, bone transplantation, congenital pseudarthrosis, mesenchymal stem cells, osteogenesis, stem cells, tibial fracture, tissue engineering

## Abstract

**Background:** NVD003 is an autologous, adipose tissue-derived stem cell-based tissue-engineered bone graft substitute with pro-osteogenic, anti-resorptive, and pro-angiogenic properties. Here, we describe highlights from the NVD003 preclinical development program as well as early clinical experience. **Methods:** NVD003 is produced in a Good Manufacturing Practice-controlled process from adipose stem cells collected during a minimally invasive liposuction procedure. The final implant is a ready-to-use moldable putty with fixed mineral content and predefined physiologic ranges of osteogenic cells and bioactive growth factors. Preclinical pharmacology studies were conducted in nude rats using a paravertebral implantation model, and subsequently, in a femoral critical-sized bone defect (CSBD) model. In a first-in-human Phase 1b/2a study, NVD003 was used for fracture osteosynthesis with classical fixation material in nine adults with recalcitrant lower limb non-union. NVD003 was also used at the discretion of treating physicians in four pediatric patients surgically treated for congenital pseudarthrosis of the tibia (CPT) with the Masquelet technique. Efficacy was evaluated as clinical healing and in terms of bone formation, bone union, and bone remodeling on radiographs and computed tomography using the extended Lane and Sandhu Scale. **Results:** Preclinical studies indicated that NVD003 requires cellularity for its bioactivity and moreover facilitates bone union when used as a graft material in femoral CSBD. In the clinical study, nine adult participants were successfully grafted with NVD003 and completed study follow-up to 24 months, with extended safety follow-up to 5 years ongoing. No adverse events were considered related to NVD003. Maximal bone formation occurred between 3 and 12 months post-implantation; the mean time to clinical healing was 6 months and the mean time to radiological union was 17 months. Ultimately, 89% (8/9) of patients achieved bone union without refracture. All four pediatric patients with CPT also achieved lasting bone union following grafting with NVD003. No safety signals were observed over a mean follow-up of 62.1 months. **Conclusions:** NVD003 represents a safe, autologous bone graft substitute product without side effects of heterotopic ossification or bone resorption. NVD003 facilitated bone union in adult and pediatric patients even under severe pathophysiological conditions.

## 1. Introduction

Achieving bone union following a large bone defect related to non-healing fracture or pseudoarthrosis, tumor resection, bone infection, or congenital abnormalities in skeletal development is highly challenging in orthopedic surgery. Patients with critical-sized bone defects often experience extensive disability and decreased quality of life due to repeated surgeries and even amputation when surgeries fail or when a defect is deemed irreparable [1]. The treatment of these defects requires some form of grafting or synthetic bone filler in addition to mechanical stabilization to facilitate bone union [1,2]. To date, the golden standard for filling critical-sized defects involves autologous bone grafting, most commonly by harvesting large quantities of healthy bone from the iliac crest or the intramedullary canal of long bones, to facilitate bone union [2]. In many cases, bone-promoting adjuvants such as recombinant bone morphogenic protein (e.g., rhBMP-2, -7) have been combined with autograft, especially in femoral and tibial non-union, in an effort to improve rates of non-union and surgical revision [3]. However, autologous bone grafting has several important limitations, including the limited nature of resources available from different bone graft harvesting sites and minor and major morbidities at donor sites, with major complications including herniation through massive bone graft donor sites, vascular injury, deep infections at the donor site, neurologic injury, deep hematoma formation requiring surgical intervention, and iliac wing fractures [4]. Autograft failure rates range from about 10 to 30% or greater depending on the specific use [4], and moreover it is generally understood that some large defects are simply not candidates for repair given the limited quantity of healthy autologous bone available for harvesting. The need for effective bone graft substitutes to facilitate the remodeling and repair of native bone tissues is a clear unmet need in orthopedic surgery.

Adequate bone healing requires a combination of physiological and biomechanical factors that promote bone regeneration. Any disturbances in these factors can lead to unfavorable bone healing conditions and inadequate bone union. The “diamond concept” is a critical framework for understanding the minimal requirements for bone healing that defines five key factors (in addition to host factors) for bone regeneration: mechanical stability, osteogenic cells, osteo-inductive factors, osteo-conductive matrix, and vascularity [5]. In severe pathophysiological conditions (e.g., hypoxia, lack of mineralized callus formation, bone resorption, and low osteogenicity), such as those found in recurrent osseous nonunion after failed conventional surgery and in some congenital conditions, inherent bone healing processes require additional support and graft integration becomes increasingly unreliable [6]. Autograft alone does not meet all five of these criteria, and similarly, most synthetic bone substitutes and ceramics lack osteoinductive and other qualities to promote bone healing in difficult environments. Promising bioprinting innovations have been combined with growth factors for bone defect filling in a periodontal setting, but remain limited by suboptimal mechanical properties and cost-effectiveness [7]. Other tissue-engineered products such as semi-solid hydrogels offer the ability to carry bone-promoting bioactive factors, drugs, or cells, but can have limited mechanical stability and issues related to biocompatibility and immunogenicity [8,9].

**Figure 1:**
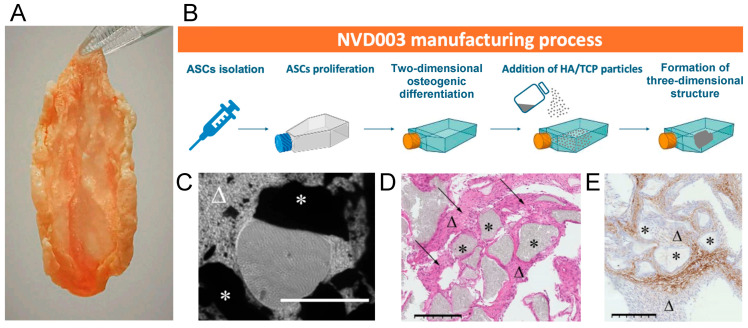
Manufacturing and characterization of the NVD003 implant. (**A**) Macroscopic view of ready-to-implant NVD003. (**B**) Manufacturing process of NVD003. Osteogenic differentiation of isolated and expanded adipose-derived mesenchymal stem cells (ASCs) is induced by culture medium supplementation with osteogenic factors, which elicits cells to secrete an extracellular matrix. Cells are then co-incubated with hydroxyapatite/beta-tricalcium phosphate (HA/TCP) particles to further potentiate the osteogenic cell phenotype and collagen synthesis, leading to the entrapment of the cells and particles in a partially mineralized extracellular matrix in the form of a three-dimensional, whitish moldable patch of putty-like material for implantation. (**C**) Phase contrast microscopy 3–7 days after the addition of HA/TCP particles showing their integration into the extracellular matrix secreted by the cells (bar = 1000 μm). (**D**) Hematoxylin–eosin staining of the final product, 8 weeks after the addition of HA/TCP particles (original magnification ×10; bar = 250 μm). (**E**) Osteocalcin staining of the final product, 8 weeks after the addition of HA/TCP particles (original magnification ×10; bar = 250 μm). * indicates HA/TCP particles; Δ indicates extracellular matrix; and arrows indicate adipose-derived cells.

NVD003 is an autologous, ready-to-use, tissue-engineered osteogenic implant with an endogenous/biogenic scaffold (i.e., free of exogenous scaffolding) for use during elective bone reconstructive procedures in place of other bone graft materials. The implant is manufactured through a proprietary, Good Manufacturing Practice (GMP)-controlled process using human stem cells isolated from a small volume of subcutaneous adipose tissue, obtained during a minimally invasive lipoaspiration procedure. Briefly, autologous osteogenic cells are obtained after ex vivo isolation, expansion, and differentiation of multipotent human adipose-derived mesenchymal stem cells (ASCs; Figure 1). Osteogenic differentiation is induced by culture medium supplementation with osteogenic factors, which elicits cells to secrete an extracellular matrix (ECM). Cells are then co-incubated with hydroxyapatite/beta-tricalcium phosphate (HA/TCP) particles to further potentiate the osteogenic cell phenotype and collagen synthesis. This leads to progressive and complete entrapment of the cells and the HA/TCP particles in partially mineralized ECM in the form of a three-dimensional moldable putty-like material for implantation. The manufacturing process requires 9–13 weeks and produces at least 20 cc and up to or exceeding 30 ccs of implant volume from a minimum of 2 cc adipose tissue collection.

NVD003 was specifically developed to facilitate bone healing in severe pathophysiological conditions (e.g., hypoxia, lack of mineralized callus formation, bone resorption, and low osteogenicity) as found in recurrent osseous nonunion after failed conventional surgery. Herein, we describe the highlights from the preclinical development program as well as the first-in-human studies, including a Phase 1/2 study in adults with bone non-union and four pediatric cases of congenital pseudarthrosis treated under the Compassionate Use designation in Belgium.

NVD003 is still under development and has not been authorized in any country inside or outside the EU for any indication at this time. NVD003 has been granted Orphan Drug Designation (ODD) for congenital pseudarthrosis of long bones in the EU (EU/3/24/2912, EMA/OD/0000158981) and US (DRU-2020-7612), Rare Pediatric Disease Designation (RPD-2020-356), and Fast Track Designation, as well as Regenerative Medicine Advanced Therapy (RMAT) designation by the United States Food and Drug Administration (FDA) for the treatment of congenital pseudoarthrosis of the tibia (CPT).

## 2. Materials and Methods

### 2.1. Animals and Experimental Design

Nude (Hsd: RH-Foxn1^rnu^) rats (6–7 weeks of age upon arrival, 9 weeks at the start of the study) were used in preclinical studies, including 11 rats for the bioactivity study and 56 animals for the CSBD study. In the bioactivity study, all animals were implanted with both treatments (whole NVD003: right side and decellularized NVD003: left side). In the CSBD study, animals first received surgery to induce the CSBD model and were then randomly divided into two treatment groups (NVD003 or HA/TCP particles alone). Animals were group-housed (when possible) in a climate-controlled facility on a 12 h light/dark schedule with chow and water available ad libitum in accordance with facility standard operating procedures and international ALAAC guidelines. Whole and decellularized NVD003 were provided by Novadip Biosciences SA (Belgium).

Briefly, decellularized NVD003 was prepared by successive incubation of the implant substance as follows, with washing in PBS for 30 min at 2–8 °C between each incubation: hypotonic solution for 24 h at 2–8 °C, hypertonic solution for 24 h at 2–8 °C, nuclease solution for 6 h at room temperature, and finally, a Trisma HCl-Triton X-100 solution for 24 h at 2–8 °C. Decellularized samples were then washed in PBS for 72 h at 2–8 °C to eliminate reagent residue. Decellularization was confirmed using manufacturing characterization procedures.

#### 2.1.1. Lumbar NVD003 Implantation

The lumbar implantation of whole and decellularized NVD003 was performed under anesthesia. For each animal, bilateral longitudinal skin incisions were made along the rachis at the lumbar level. Muscular stalls were established into the lumbar muscles, cauterized, and whole and decellularized NVD003 were implanted on the right and left sides, respectively. A volume of 0.3 cc of each item was implanted at each site, corresponding to 500 mg or 2.45 × 10^6^ cells of NVD003 or 500 mg of decellularized NVD003, respectively. The muscle and skin were sutured using absorbable surgical thread and animals were allowed to recover in a dedicated area before being returned to their home cage. Clinical follow-up included daily checks for viability, general condition, and mobility; body weight measurement; and imaging procedures on days 3 (D + 4) and 28 (D + 29) after implantation. Animals were euthanized on D + 30 for necropsy and the explantation of the muscle implant sites for histology analysis (5 explant samples per treatment) and qRT-PCR analysis (10 explant samples per treatment). One animal was excluded from the explant analysis. A complete description of study procedures is available upon request.

#### 2.1.2. CSBD Model Implantation of NVD003

The CSBD model was induced under isofluorane anesthesia using the RatFix System (RISystem) procedure. Each animal received a bone defect of 5 mm using previously described methods [10]. Post-surgery, animals were housed individually and received 0.05 mg/kg/day buprenorphine HCl for two days for pain management. Three weeks after CSBD induction, animals were imaged (2D-μCT) to confirm successful model induction and those eligible for implantation were subsequently randomized (based on weight body repartition criteria) to receive implantation with NVD003 or HA/TCP control. Briefly, the implantation site was prepared and a volume of approximately 0.313 cc NVD003 (corresponding to 500 mg or 7.29 × 10^6^ cells) or approximately 0.344 cc HA/TCP particles (corresponding to 500 mg) was implanted depending on the group assignment. The implantation volume was sufficient to fill at least the missing bone segment. The muscle and skin were sutured using absorbable surgical thread and animals were allowed to recover in a dedicated area before being returned to their home cage. Once again, animals received 0.05 mg/kg/day buprenorphine HCl for two days for pain management. Forty-four animals were included following the second surgery. Clinical follow-up included daily checks for viability, general condition, and mobility. Body weight was measured twice weekly and imaging procedures (3D-μCT) were performed for 7 rats/group prior to euthanasia at 4, 8, and 12 weeks after implantation. Animals were euthanized the day after 3D-μCT imaging at their respective time point by lethal intraperitoneal injection of pentobarbital for necropsy and explantation of the implanted femur. For each time point, 3 explants/group were processed for histology and 4 explants/group for qRT-PCR (weeks 4 and 8 only). A complete description of study procedures is available upon request.

### 2.2. NVD003-CLN01 Study Design and Participants

Study CLN01 was a prospective, single-arm clinical study conducted across 5 centers in 2 countries: Belgium (CHU Ambroise Paré Mons, CHIREC Brussels, GHDC Charleroi, and CHU Brugmann Brussels) and Luxembourg (CHU Centre Hospitalier de Luxembourg). Eligible patients were ≥ 18 years old; had confirmed non-union of a single metaphyseal or diaphyseal bone defect in the lower limb with a maximum size of 4 cm; had normal or low bone density (bone mineral density T-score > −2.5); and were not taking bisphosphates or other bone-modulating agents at the time of enrollment. Baseline characteristics recorded for each participant included age, weight, smoking status and comorbidities, primary fracture date, primary facture location and type, and defect size. The Non-Union Severity Score was used to quantify baseline severity of the non-union based on multiple bone parameters, soft tissue, and patient characteristics [11]. Written informed consent was obtained prior to study participation. The study protocol (EudraCT 2018-000299-13/EU CT 2024-517762-42-01/NCT06335394) was approved by institutional ethics committees in Belgium, Luxembourg, and Switzerland.

Enrolled participants underwent a minimally invasive subcutaneous liposuction procedure under local or general anesthesia to harvest fatty tissue for the generation of autologous osteogenic cells and subsequently production of the NVD003 implant in a GMP-controlled process. Briefly, adipose tissue was subjected to enzymatic digestion and ex vivo expansion of adipose tissue-derived mesenchymal stem cells. Cells were differentiated in osteogenic medium and HA/TCP was added to trigger the secretion of an extracellular matrix and the formation of the three-dimensional osteogenic implantable putty over an 8-week period. The final implant is sterile, produced in large volumes, does not require any additional manipulation before application, and is easily moldable to fill and encapsulate large bone defects. The dosage basis for NVD003 is volume in cubic centimeters (cc). Implants for human use met the acceptance criteria in Table 1.

Approximately 12 weeks after the liposuction procedure, participants underwent a standard-of-care, non-restricted bone reconstructive surgery using NVD003 to fill the bone void. NVD003 was used as a single agent without adjunctive autologous bone grafts (e.g., iliac crest bone grafting [ICBG]) or other bone-enhancing agents (e.g., recombinant human bone morphogenic protein-2 [rhBMP-2]). For one patient, adipose tissue collection was performed at the time of the first phase of the Masquelet technique and the graft was implanted during the second phase [12].

#### Study CLN01 Endpoints and Assessments

The primary endpoint was safety, assessed as adverse events (AEs) occurring from the time of the liposuction procedure to 24 months after the grafting surgery. All AEs were rated for seriousness, severity, expectedness, and relatedness to both procedures (liposuction and grafting surgery) or to NVD003.

Secondary endpoints included radiographic efficacy assessed at 3, 6, 9, 12, 15, 18, 21, and 24 months. Images were assessed by two independent senior consultant expert musculoskeletal radiologists. All post-implantation X-ray images (6 weeks and 3, 6, 12, 15, 18, 21, and 24 months) and computed tomography (CT) images (6, 12, and 24 months) were evaluated using an extended Lane and Sandhu Scale (eLSS, [13]) that rates bone formation (longitudinal filling, 0–5), bone union (transverse filling, 0–5), and bone remodeling (continuity of bone architecture, 0–2). Disagreement between readers was resolved by joint adjudication. Clinical healing was defined as clinical and radiographic progression towards healing over 3 consecutive months on serial radiographs and a minimum of 9 months after the first attempt of surgical bone repair, or a minimum of 6 months after the second (or any subsequent) attempt of surgical bone repair/union as assessed by the investigator using the eLSS. Per local regulations in Belgium and Luxembourg, participants are followed for a total of 5 years post-implantation for safety data collection.

### 2.3. Statistical Analysis

Data were summarized descriptively: continuous variables were summarized as the mean ± standard deviation or median (range); categorical variables were summarized as the number and percentage. The safety population included all participants who signed informed consent, whereas the efficacy population included participants who underwent the grafting procedure. Time to clinical healing and time to bone union on plain X-ray were analyzed using Kaplan–Meier survival curves. Inter-reader variability on the eLSS was determined using the weighted kappa method of Fleiss et al. [14]. All statistical analyses were performed using IBM SPSS Statistics (version 21.0 or higher; IBM Corporation, Armonk, NY, USA) and StatXact (Cytel, Cambridge, MA, USA).

### 2.4. Compassionate Use Program Implantation of NVD003

Four pediatric patients with CPT were treated using NVD003 as the grafting material in the second stage of the Masquelet technique at the request of the treating physician and under specific provisions of the Belgian legislation (Urgent Medical Need and Hospital Exemption programs). Case narratives are provided for each patient and patients were followed for a mean period of 62.1 months (range, 45.5–84.2) post-implantation. Efficacy was assessed in terms of investigator-assessed clinical healing (clinical deterioration, clinical status quo, positive clinical progression, or fully clinically healed), weight bearing and walking status (no weight bearing, toe-touch weight bearing, partial weight bearing, full weight bearing, or full weight bearing with walking), and local radiological evaluation (not evaluable, deteriorated radiological healing, status quo, positive radiological healing, or full radiological healing) at each visit.

## 3. Results

### 3.1. Manufacturing and Characterization of NVD003

NVD003 is manufactured from a small quantity (5–10 cc, minimum 2 cc) of adipose tissue (Figure 1A) obtained through a small-volume minimally invasive liposuction procedure. Manufacturing of the implant requires a period of 9–13 weeks and the final implant is provided ready-to-use in five 250 mL containers including the implant materials shipped in CMRL-1066 culture transport medium. The final NVD003 drug product is a moldable putty containing approximately 14 × 10^8^ viable autologous osteogenic cells and 11.00 g ± 10% HA/TCP particles embedded in partially mineralized extracellular matrix (Figure 1B–E). The osteogenic potential of the final NVD003 implant is GMP-controlled, driven by a fixed mineral content and predefined physiologic quantity ranges of active osteogenic cells and osteogenic and angiogenic growth factors. The exact specifications of each batch are tested and compliant with specific manufacturing acceptance criteria, which confirm product appearance, cellularity and cell viability, and the presence and quantities of growth factors osteoprotegerin (OPG), insulin-like growth factor-1 (IGF-1), and vascular endothelial growth factor (VEGF; Table 1). Additional non-proprietary information regarding the production and characterization of NVD003 is available upon request.

### 3.2. Preclinical Development of NVD003

**Figure 2:**
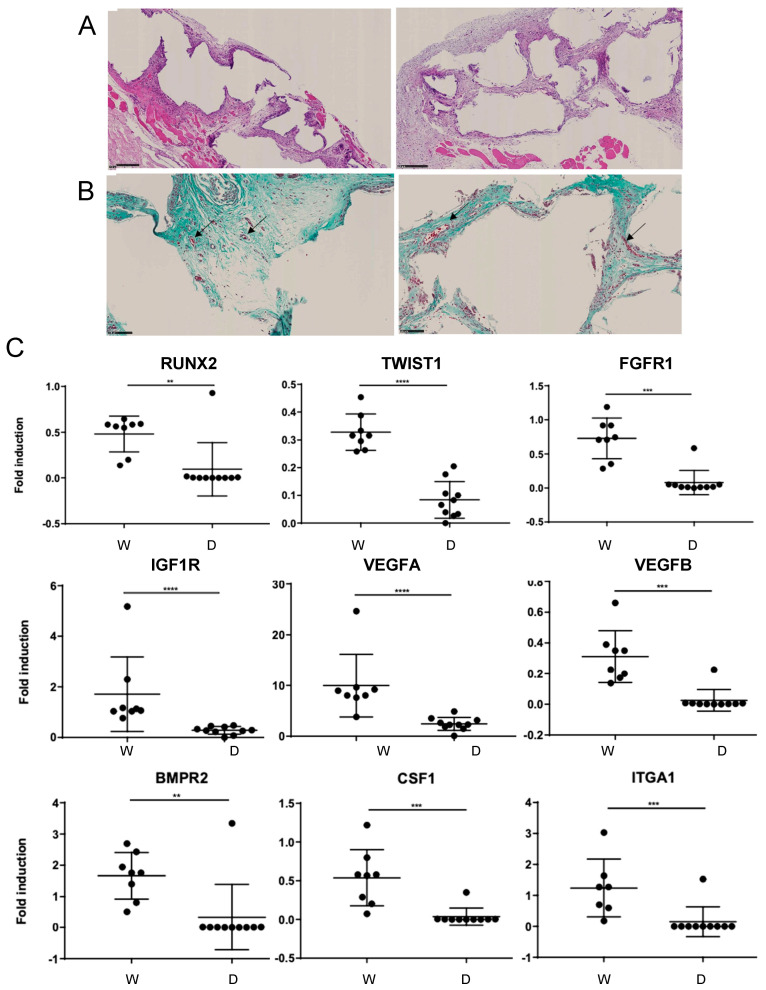
Characterization of 30-day whole versus decellularized NVD003 explants in nude rats. (**A**) Representative hemoxylin and eosin staining and (**B**) Masson’s trichrome staining (vascularization, arrows indicate blood vessels) of NVD003 (**left**) and decellularized NVD003 explants (**right**) 30 days after implantation. (**C**) Modulated genes related to skeletal development in 30-day explants. W, whole NVD003; D, decellularized NVD003; N = 8–10; and ** *p* < 0.01, *** *p* < 0.001, and **** *p* < 0.0001.

Batches of NVD003 meeting the acceptance criteria were evaluated as part of the preclinical development program, which included both pharmacology studies and toxicology studies with tumorigenicity and biodistribution components (data available upon request). Briefly, the pharmacological activity of NVD003, and specifically, the role of viable osteogenic cells contained within the implant were initially evaluated in a comparison of whole or decellularized NVD003. Whole or decellularized NVD003 was implanted into the muscle of the lumbar paravertebral region of nude rats and explanted 30 days thereafter for qRT-PCR and histological analyses. Notably, decellularized NVD003 did not meet the manufacturing acceptance criteria and had a cellularity of 6 cells/mm^3^ (versus 163 cells/mm^3^ prior to decellularization) and, consequently, almost complete depletion of growth factors as assessed by ELISA including a 4-fold reduction in IGF-1 (5 ng/g of NVD003) and quantities of OPG and VEGF below the lower limit of detection. In whole 30-day explants, there was no difference in vascular area between groups (1.1 ± 0.2 vs. 1.9 ± 0.6, *p* = 0.8, n = 5; representative images in Figure 2A,B). In contrast, qRT-PCR of explant tissues revealed a significant enhancement in the expression of osteogenic genes (RUNX2, TWIST related protein 1, Type 1/2 BMP, EGFR, CSF-1, FGF/FGFR, IGF1R, VEGF, and ITGA1) compared to decellularized NVD003 explants (see Figure 2C; additional data available upon request), confirming the role of product cellularity and the bioactivity of secreted factors.

**Figure 3:**
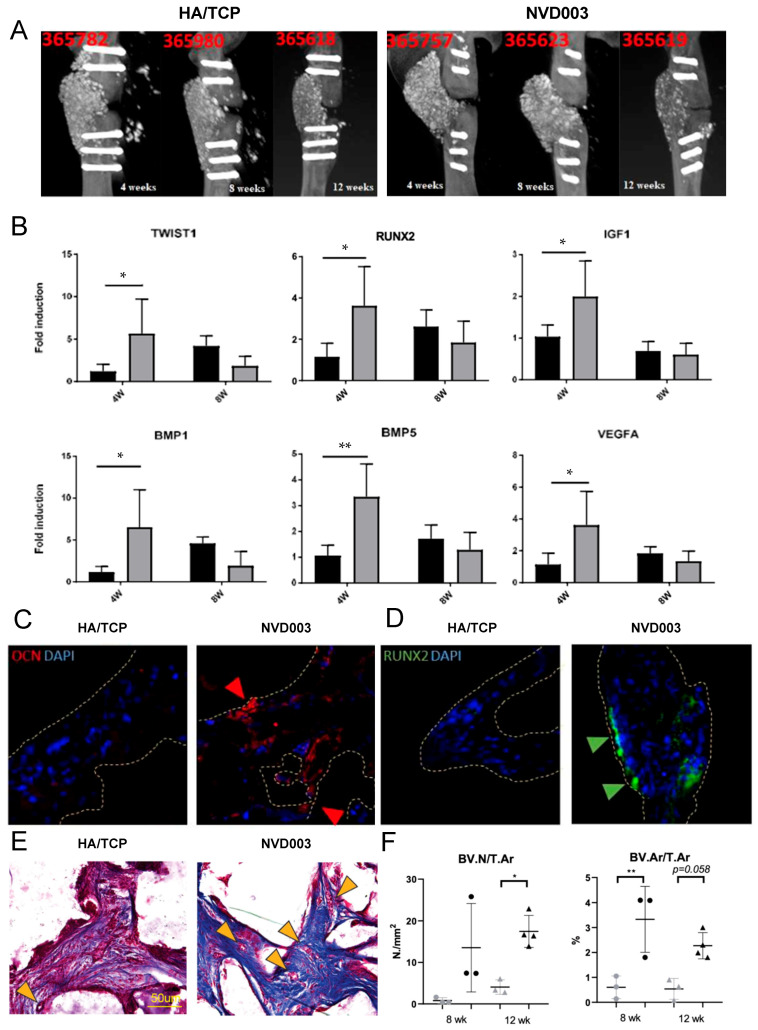
NVD003 implantation in a nude rat model of critical-sized bone defect. (**A**) Representative μCT images of the implantation site with HA/TCP particles (**left**) or NVD003 (**right**) at 4, 8, and 12 weeks post-implantation. (**B**) qRT-PCR of HA/TCP (black) and NVD003 (grey) explants at 4 and 8 weeks post-implantation, evaluating the induction of osteogenic genes (N = 4 per timepoint). (**C**) Representative osteocalcin (OCN) and (**D**) RUNX2 immunohistochemistry of HA/TCP (**left**) and NVD003 (**right**) explants at 12 weeks post-implantation. Graft material is outlined highlighted with dashed yellow lines. Increased expression of OCN and RUNX3 in within the graft area is evidenced by red and green arrows, respectively. (**E**) Representative histology of HA/TCP (**left**) and NVD003 (**right**) explants at 12 weeks post-implantation. An increase in small caliber blood vessels (yellow arrows) is noted. (**F**) Blood vessel number/tissue area (**left**) and blood vessel area/tissue area (**right**) for HA/TCP (grey) and NVD003 (**right**) explants at 8 and 12 weeks post-implantation. N = 3 per timepoint; * *p* < 0.05 and ** *p* < 0.01 vs. HA/TCP at the corresponding time point.

Subsequently, NVD003 implantation was compared to implantation of HA/TCP particles alone in an orthotopic nude rat model of femoral critical-sized bone defect (CSBD). Four weeks after the induction of femoral CSBDs and the placement of an internal fixation RatFix System, animals without spontaneous consolidation were implanted with NVD003 or HA/TCP particles alone. Animals were evaluated at 4, 8, and 12 weeks post-implantation, including explantation for molecular and tissue analyses. μCT imaging at each time point confirmed implant stability, sustained mineralization, and the absence of resorption over time, and moreover, NVD003 was associated with better defect filling (Figure 3A). NVD003 was associated with an increase in the expression of osteogenic and angiogenic genes at 4 weeks but not 8 weeks post-implantation, signaling an early osteogenic response (Figure 3B). Consistent with this observation, immunohistochemistry identified significant increases in RUNX2 and OCN, indicative of osteogenic differentiation, in NVD003-implanted tissues at 12 weeks (Figure 3C,D), and increased graft vascularization (the number of vessels per area and vascular area) at 8 and 12 weeks, compared to HA/TCP particles alone (Figure 3E,F). No apparent signs of toxicity were observed in this study.

The results of the toxicology program showed no noteworthy toxicity or tumorigenicity for NVD003. Similarly, cells contained in the NVD003 product were not found to distribute outside of the implantation site nor result in ectopic tissue formation 30 days post-implantation. Data from these studies are available upon request. Overall, the conclusions of the preclinical development program for NVD003 supported the posited mode of action and safety extrapolations supported initial in-human use of up to 31 cc NVD003 in pediatric or adult patients.

### 3.3. NVD003 in Adults with Recalcitrant Long Bone Non-Union

Fracture complications and particularly long bone non-union (pseudarthrosis) remain important challenges for orthopedic surgeons. Bone non-union is defined as the absence of clinical and radiographic progression towards healing over 3 consecutive months on serial radiographs and a minimum of 9 months after the first attempt of surgical bone repair, or a minimum of 6 months after the second (or any subsequent) attempt of surgical bone repair [5,15]. To this end, post-traumatic recalcitrant lower limb non-union in adults was selected as the target indication for a first-in-human study of NVD003. This Phase 1b/2a study was a prospective, single-arm clinical study conducted across five centers in Belgium and Luxembourg. Of eleven participants (eight in Belgium and three in Luxembourg) enrolled between August 2018 and January 2020, one participant was not grafted due to a non-eligibility criterion and one participant withdrew from the study due to liposuction-related pain. Baseline characteristics and fracture details for the nine remaining participants (four men and five women; median age, 56 [21–74] years) are summarized in Table 2. Target bone defects included four tibial and five femoral traumatic fractures.

NVD003 implant production was successful for all nine participants from a mean volume of 9.0 ± 1.49 mL fatty tissue. The mean duration of reconstructive surgery was 149 ± 92 min (median, 142 [39–259] min) and the mean applied NVD003 graft volume was 15.6 ± 2.0 cc (median, 16.5 [11.4–17.9] cc). No intraoperative complications or blood transfusions were reported.

#### 3.3.1. Efficacy

**Figure 4:**
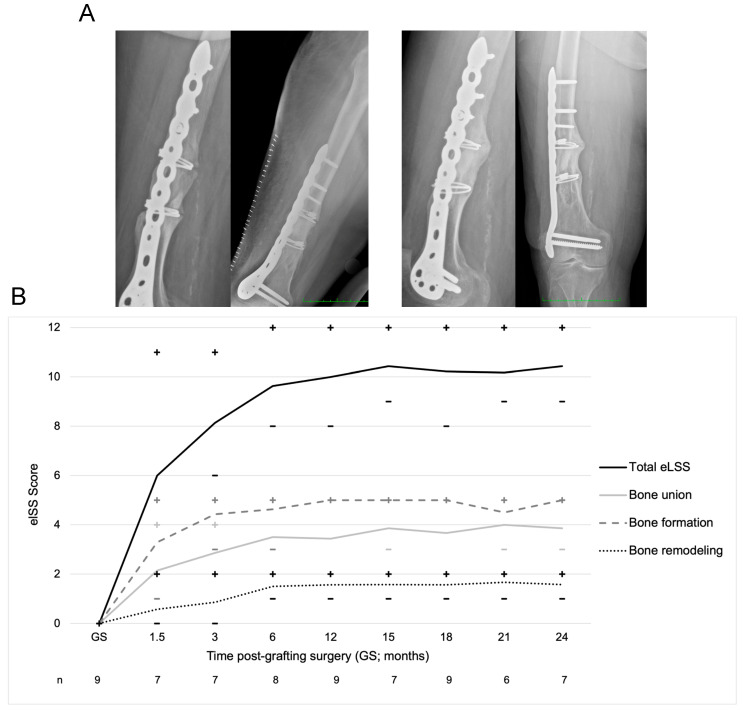
Representative images of NVD003 implantation and summary of eLSS scores from X-ray in Study CLN01. (**A**) Representative radiographic images of participant PABE301 immediately post-implantation (**left**) and at 24 months post-implantation demonstrating bone union (**right**). (**B**) Mean Extended Lane and Sandhu Scale (eLSS) total and subscores for X-ray images following grafting surgery. The total eLSS score is the sum of three individual components: bone formation (longitudinal filling, 0–5), bone union (transverse filling, 0–5), and bone remodeling (continuity of bone architecture, 0–2). Minimum (–) and maximum (+) values are denoted above and below the plotted data line in corresponding colors.

The eLSS was used to evaluate bone formation, bone union, and bone remodeling on X-rays and CT images due to the high radiopacity of the NVD003 implant, which impedes the direct evaluation of cortical continuity, callus formation, and remodeling early after implantation. The presence of early bone formation was confirmed for all patients on X-ray at 6 weeks post-implantation and the first significant bony bridging signals (defined as an eLSS union score of 3/5 or 50–75% filling of the transverse gap against baseline) were detected in all patients within the first 3 to 12 months. Individual eLSS subscores from X-ray are tabulated in Supplementary Table S3 and the progressions of mean eLSS subscores and total eLSS score over time are visualized in Figure 4 (see also Supplementary Table S4). Maximal eLSS bone formation subscores were recorded in one participant at 6 weeks, three participants at 3 months, two participants at 6 months, and three participants at 12 months post-implantation. Increases in bone union of up to 75% (eLSS score 3/5) were documented in seven participants at 6 months and in all nine participants at 12 months post-implantation. At 18 months, five participants achieved a 100% increase in bone union. At 24 months, the mean and median total radiographic eLSS scores were 10.43 and 10.00 (maximum score 12), respectively (Figure 4B). CT findings demonstrated a similar trend in healing: the mean and median total CT eLSS scores at 24 months were 8.57 and 8.00, respectively (Supplementary Figure S1). High inter-rater reliability was reported for both radiographic and CT eLSS scoring (weighted kappa 0.73–0.95, good or excellent). The mean and median times to radiological union on X-ray as determined by Kaplan–Meier survival analysis were 17 months and 18 months, respectively (standard error, 2.83; lower bound, 9.24; and upper bound, 26.77).

The mean and median times to clinical healing (defined in the Methods) as determined by Kaplan–Meier analysis were 6 months and 9.17 months, respectively (standard error, 4.5; lower bound, 0.00; and upper bound, 14.77). The rate of clinical healing at 24 months (evaluable in nine participants) was 89% (eight of nine). One tibial refracture was reported: despite early callus formation with no signs of resorption, a tibial screw broke 6 months post-GS in participant PABE401. Seventeen months post-GS, the complete tibial plate was removed, which induced fibular refracture and increased instability to the operated area. The NVD003 graft was removed due to nonunion and the patient underwent revision surgery. Failure likely resulted from a combination of factors including fracture location and inability to achieve anatomical alignment leading to fracture instability and the failure of the orthopedic fixation materials. This patient did not achieve clinical healing or second radiological union by the end of the study period.

#### 3.3.2. Safety

In nine participants grafted with NVD003, 51 treatment-emergent AEs were reported over the complete study duration and are summarized in Supplementary Table S1. Of these, 15 AEs were rated as serious and none were considered related to NVD003. AEs were scored as mild (n = 27, 53.0%), moderate (n = 8, 15.7%), or severe (n = 16, 31.4%). Three patients reported mild to moderate pain during or after the liposuction surgery performed under local anesthesia; thereafter, prophylactic pain medication and general anesthesia were introduced to prevent additional liposuction-related occurrences of pain in the remaining six patients. The resorption of NVD003 was initially suspected in one case, supported by a delineated radiolucent area within the bone graft; however, after additional follow-up, the radiolucent spot disappeared, indicating normal bone remodeling activity. The results of this study indicate an 89% rate of bone union without refracture and support the safety of the NVD003 implant for treating recalcitrant bone non-union.

#### 3.3.3. Long-Term Follow-Up

Of nine participants who completed active follow-up, one did not sign the LTFU consent form, one participant withdrew consent, and one participant died during LTFU (unrelated cause). Therefore, six participants have been included in the LTFU study. To date, four participants have completed LTFU and follow-up is ongoing in the remaining two participants. LTFU up to 5 years post-implant has not revealed any safety concerns or instances of refracture to date.

### 3.4. NVD003 in Pediatric Patients with Congenital Pseudarthrosis of the Tibia

CPT is a rare pediatric disease that is present from birth and ultimately leads to the development of a non-healing fracture (pseudarthrosis) of the tibia. In up to 84% of cases, CPT is related to neurofibromatosis-1 (NF1), a clinically diagnosed multisystemic neurocutaneous disease caused by loss-of-function variants in the *NF1* gene [16]. Although the clinical expression of NF1 is highly variable and its relationship with CPT remains unclear, non-NF1-CPT and NF1-CPT appear to have similar clinical features and outcomes of surgical treatment [17], and the presence of NF1 did not influence the efficacy of the NVD003 graft in preclinical development (data available upon request).

Following initial fracture, most patients with CPT require repeated surgical interventions to achieve long-term union of the tibia, prevent refracture, correct limb length discrepancy, avoid leg deformity and nearby joint stiffness, and preserve normal function and bone growth of the leg [18]. Nevertheless, the combined long-term success rate for SOCs is estimated to be about 51% [19,20] and failure to achieve and maintain bone union results in the amputation of the lower leg in about a quarter of patients [21]. NVD003 was specifically designed for grafting in compromised bone healing environments and was used as the grafting material in the second stage of the Masquelet technique at the request of treating physicians and under specific provisions of the Belgian legislation (Urgent Medical Need and Hospital Exemption programs) in four pediatric patients with CPT.

#### 3.4.1. Case 1

**Figure 5:**
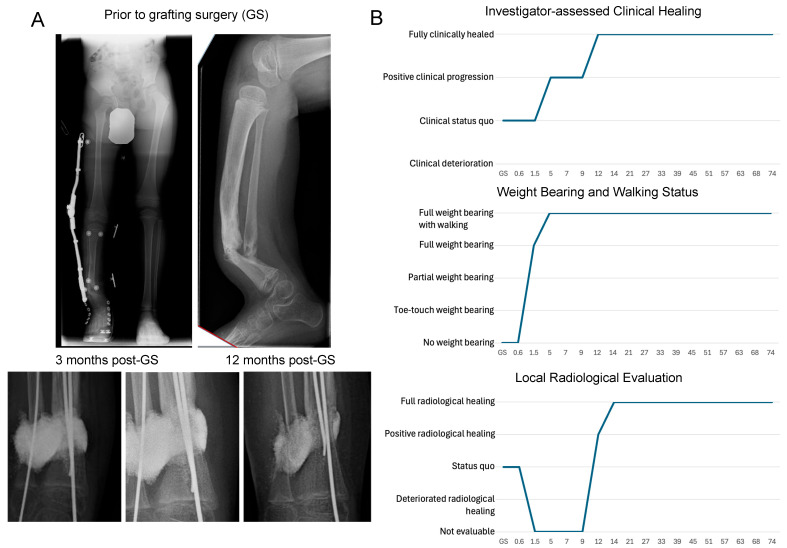
Representative pediatric case: Case 1, a 5-year-old boy with congenital pseudarthrosis of the tibia. (**A**) Baseline radiographic images of the tibial pseudoarthrosis (**top**) and images of the NVD003 implant at the defect site at 3, 12, and 27 months post-grafting surgery (GS; **bottom**) in Case 1. (**B**) Investigator-assessed clinical healing, weight bearing and walking status, and local radiological evaluation of healing over 74 months of follow-up.

A 5-year-old boy with Crawford grade IV CPT presented with a history of previous failed surgeries. Approximately 19 cc of adipose tissue was harvested from the ipsilateral leg and about 25.2 cc NVD003 was implanted on 5 January 2018. The absence of pain was noted at the first follow-up visit; full weight bearing was noted as early as 1.5 months post-GS and positive clinical progression was noted from 3 to 5 months post-GS. The clinical status of the patient, weight bearing capability, and daily activity including walking ability improved gradually over time (Figure 5). Early X-ray and CT images were not evaluable due to the density and radiopacity of the graft, although signs of healing/union were noted from 12 months and full radiological healing was observed 14 months post-GS. During the follow-up period exceeding 74 months, the treating physician reported four mild SAEs, all considered not related to NVD003 but related to the graft surgery (Supplementary Table S2).

#### 3.4.2. Case 2

A 9-year-old boy presented with Crawford grade IV, El Rossasy-Paley type 2 CPT, and a concomitant diagnosis of NF1. Adipose tissue (8.8 cc) was harvested from the contralateral leg. About 14.1 cc NVD003 was implanted on 25 June 2019. Positive clinical and radiological progression were noted from 1.4 months and partial weight bearing and signs of radiological union (up to 75% filling of the defect compared to baseline) were observed from 3 months post-GS. A lack of complete weight bearing and walking during the first 9 months post-GS were triggered by important underlying NF1 comorbidities (laryngeal neurofibroma), but the patient had full weight bearing with walking at 13 months. Full and persistent bone formation was confirmed at 6 and 13 months.

Clinical deterioration was observed at 19 months and the patient was hospitalized for a revision of the fixation materials; thereafter, the patient had no pain and continued to show positive radiological healing, with full radiological healing observed from 26 months post-GS. During follow-up to 66 months, the treating physician reported four mild to moderate (S)AEs, all considered not related to NVD003 (Supplementary Table S2).

#### 3.4.3. Case 3

A 3-year-old boy presented with Crawford grade IV and concomitant diagnoses of glucose 6 phosphate-dehydrogenase deficit and NF1, clinically characterized by Café-au-lait spots and hypersignal lesions in the cerebellum and the bilateral supratentorial region. Adipose tissue (5.6 mL) was harvested from the ipsilateral leg. About 19.6 cc of NVD003 was implanted on 8 September 2020. No post-surgical complications were reported. The patient had no pain and showed positive clinical progression from his first follow-up after surgery, with full weight bearing and walking as well as positive radiological healing from 1 month post-GS. Full clinical healing was documented from 4 months and full radiological healing from 17 months post-GS. During follow-up to 48 months, the patient experienced one AE of distal migration of the fibular nail that was considered not related to NVD003 (Supplementary Table S2).

#### 3.4.4. Case 4

A 16-year-old girl presented with congenital pseudarthrosis of the right tibia and no other comorbidities. The patient complained of intermittent leg pain that prevented her from walking long distances. About 6.4 mL of adipose tissue was harvested and approximately 23.1 cc NVD003 was implanted on 8 March 2020. The patient had no pain and showed positive clinical and radiological progression from her first follow-up after surgery. At 6 weeks, the patient had full clinical healing and full weight bearing and walking. One AE of distal migration of the tibial nail and screw breakage was considered unrelated to NVD003 and surgery to remove the screw was performed at 12 months; at this time, full radiological healing was observed and persisted through the follow-up period. At 32 months, the patient was walking without any brace or protection. No other AEs were reported up to 45 months post-GS (Supplementary Figure S4).

## 4. Discussion

NVD003 is a first-in-class bioengineered autologous implant and bone graft replacement that harnesses the potential of mesenchymal stem cells for bone repair in patients with large-scale defects [22] without the need for combination with supraphysiological quantities of growth factors and adjunctive scaffolding. The small-volume liposuction procedure required for harvesting stem cells poses less patient burden compared to other stem cell harvesting methods (e.g., from bone marrow) and is considerably safer than autologous bone harvesting.

The composition of NVD003 includes key bioactive factors that support bone formation and union: OPG is a factor that critically regulates bone resorption as a decoy receptor for RANKL, inhibiting the differentiation of osteoclasts and thereby promoting osteoblast activity [23]. IGF-1 promotes the osteodifferentiation of mesenchymal stem cells and plays a key role in bone mass acquisition and maintenance [24,25]. VEGF, in addition to its role in angiogenesis, plays a critical role bone repair, particularly during callus formation and bone remodeling [26]. Based on the known roles of these factors, the composition of NVD003, and the activity of NVD003 in preclinical studies, the mode of action is presumed to be related to (i) the promotion of osteogenesis and angiogenesis through ASC-secreted growth factors, (ii) the secretion of antiresorptive factors by ASCs that limit osteoclast activity, and (iii) a high degree of mineralization contributing to the direct formation of a hard bone callus.

The research described herein summarizes the first-in-human experience with NVD003 in a Phase 1b/2a clinical study of adult recalcitrant lower limb non-union and in four pediatric Compassionate Use cases treated under specific provisions of the Belgian legislation. In the Phase 1b/2a clinical trial, NVD003 implantation was not causally implicated in any of the recorded AEs; did not produce any foreign body responses; and did not cause any unexpected delayed adverse events per long-term follow-up data available to date. As previously described for scaffold-free tissue engineered products [27], the autologous nature of the harvested adipose stem cells and the absence of any artificial or exogenous scaffold precludes the risk of disease transmission and drives the high biocompatibility of NVD003. The risk of infection at the implant site is reduced by guaranteeing the sterility of the NVD003 product and by omitting the autologous bone harvesting step, reducing operative time and the number of perioperative preparatory handlings (e.g., bone drilling, grinding, and mixing). Risks associated with the adipose tissue harvesting procedure were also low, in part due to its minimally invasive nature. In this study, the use of prophylactic pain medication and general anesthesia during harvesting resolved the primary complaint of pain at the harvesting site.

Regarding efficacy in the clinical study, NVD003 was associated with a rate of clinical healing without refracture of 89% (eight of nine participants), with the first significant bony bridging signals detected in all patients within the first 3 to 12 months. These rates are comparable to and in some cases exceed the performance of autograft bone in similar indications with or without the addition of bone-promoting adjuvants [28,29,30]. There were no instances of heterotopic ossification or NVD003 resorption. In four pediatric patients grafted with NVD003, none of the mild to moderate AEs reported were classified as related to NVD003 and all patients achieved bone union and positive functional outcomes. NVD003 accordingly showed an excellent safety profile in pediatric patients, comparable to that observed in adults, and moreover facilitated union in a pathological “worst-case scenario” characterized by compromised bone healing capacity. Achieving lasting union in CPT is notoriously difficult, especially after the failure of ICBG. The findings suggest that NVD003 grafting is a viable alternative to amputation or ICBG in these patients.

The use of the NVD003 graft is not without its challenges. Most notably, NVD003 has a production lead time of 9 to 13 weeks. This duration is compatible with multiple-step surgical strategies such as the Masquelet technique or otherwise can be considered a reasonable wait period in most cases given very low patient morbidity relative to other grafting methods, and given the fact that most patients requiring this graft are not receiving treatment in an emergency setting or have otherwise already waited months or years for treatment, or were ineligible for further surgical treatment (e.g., in the case of failed ICBG) and facing amputation. Another important challenge is related to the high radiopacity of the bone graft due to the HA/TCP component of NVD003, which complicates the ability of radiologists to visualize and document radiological union early after surgery (up to 12 months). In our study, we used the eLSS to assess radiological healing and moreover placed emphasis on clinical healing. In a real-world setting, clinical outcome measures such as weight bearing, walking, and pain are used to confirm ongoing union (rather than repeated imaging) and similarly should receive priority in the follow-up of patients treated with NVD003. Lastly, the potential cost-effectiveness of NVD003 should be evaluated on a case-by-case basis, especially when weighed against the cost of repeated failed surgeries or limb amputation over a lifetime.

The evaluation of NVD003 is ongoing and the implant is not yet approved for any indication in any country. Limitations of the development program to date are primarily related to the early nature of the development phase, specifically, the small sample size and heterogeneity in patient characteristics and surgical interventions. A formal first-in-pediatric study of NVD003 in patients with CPT is ongoing (NCT05693558) and a subsequent pivotal randomized, controlled study has been initiated (NCT06335394). Despite the limited nature of available data, no safety signals were identified in the preclinical development program or reported from the patients described herein. Overall, NVD003 exhibits excellent osteointegration and efficacy for bone defect healing in traumatic or pathological contexts.

## 5. Conclusions

The sum of available preclinical and clinical data regarding NVD003 to date corroborates its safety and osteogenic effects when used as a bone graft substitute. The efficacy data confirm that NVD003 readily osteointegrates, is not subject to resorption, and promotes union even under conditions of compromised bone healing. The absence of any short- or long-term safety signals up to 5 years after implantation and a high observed rate of union in adults, as well as the performance of NVD003 in an extreme pathological setting of pediatric CPT, support opportunities for larger scale, controlled clinical investigations of NVD003 in the future.

## Figures and Tables

**Table 1:** Acceptance criteria for NVD003 manufacturing.

Parameter	Test Method	Acceptance Criterion
Appearance	Visual Inspection	A large patch (or several smaller patches) of whitish moldable 3D putty Absence of observable foreign particulate matter
Sterility	USP<71>/Ph. Eur. 2.6.1	No growth
Mycoplasma	USP<63>/Ph. Eur. 2.6.7	<10 CFU/mL (limit of detection)
Endotoxin	USP<85>/Ph. Eur. 2.6.14	≤0.5 (endotoxin units/mL)
Quantity (Mass)	Weighing	Whole quantity (mass) of the drug substance at the time of drug product formulation: ≥26 g
Quantity (Cellular Density)	Histology	<14.5 (E07 cells/cm^3^)
Composition (Fraction of ECM + Cells, Drug Product)	Weighing	Characterization (organic fraction, %)
Viability (Apoptosis)	IHC CASP3	≤5 (% cleaved CASP-3+ cells)
Viability (Proliferative)	IHC Ki67	No specification (% Ki-67 + cells)
Viability (Resting Cells)	IHC (calculated)	≥70 (%)
Potency	ELISA VEGF	0.20–18 (pg/µg protein; secretion after 72 h)
Potency	ELISA OPG	16–455 (pg/µg protein; content after 72 h)
Potency	ELISA IGF1	0.40–15 (pg/µg protein; content after 72 h)

Abbreviations: CASP3, caspase-3; ECM, extracellular matrix; ELISA, enzyme-linked immunosorbent assay; IGF1, insulin-like growth factor 1; IHC, immunohistochemistry; OPG, osteoprotegerin; USP, United States Pharmacopeia; and VEGF, vascular endothelial growth factor.

**Table 2:** Baseline characteristics and fracture and implantation details of participants in Study CLN01.

ID	PABE102	PABE301	PABE401	PABE402	PABE501	PABE504	PALU101	PALU102	PALU103
Sex	Female	Female	Female	Female	Male	Male	Female	Male	Male
Age (year)	57	73	74	56	46	62	21	44	38
Weight (kg)	58	72	92	64	88	79	51	82	92
Primary fracture date (year)	Apr-17	Oct-16	Sep-18	Nov-18	Nov-14	Jun-16	Aug-16	Jul-13	Jan-06
Primary fracture location	Femur (left)	Femur (right)	Tibia/Fibula (right)	Tibia/Fibula (right)	Femur (Right)	Tibia (left)	Femur (right)	Tibia/Fibula (right)	Femur (left)
Fracture type	Trauma, closed high-velocity (triple) fracture	Trauma, closed low-velocity fracture	Trauma, closed low-velocity fracture (also including pelvic fracture)	Trauma, closed low-velocity fracture	Trauma, open grade IIIB/IIIC high-velocity fracture	Trauma, closed high-velocity fracture	Trauma, open grade 1 high-velocity fracture (also including rib fracture)	Trauma, open grade IIIB/IIC high-velocity fracture	Trauma, closed high-velocity fracture
Non-Union Severity Score	18	9	16	22	21	13	16	29	15
Height/Size of fracture defect (cm)	0.5–1	0.5–1	>1–≤3	>3	0.5–1	0.5–1	>3	0.5–1	>3
Number of previous interventions	1	1	3	4	8	6	1	18	4
Important medical history	Osteoporosis of the spine (2018), osteopenia of the hip (2018)			Bone resorption (2019)	Wound debridement and removal of initial bone graft (2018)	Left femoral fracture (1976), distal left tibial fracture (1980)		Osteomyelitis (2015–2017), multiple surgical site infections, device failures, and reinterventions with changes of fixation devices (2015–2017)	Osteomyelitis (2005–2019), arthrotic discopathy L4-L5 and L5-S1 (2019)
Date grafting surgery	12-Dec-18	07-Jan-19	27-Feb-20	08-May-20	15-Feb-19	20-Dec-19	16-Jan-19	04-Jul-19	15-Apr-20
Volume NVD003 used (cc)	13.7	9.3	16.5	16.8	11.4	15.8	17.9	16.6	17.2

## Data Availability

Research data reported in this study including complete datasets are available upon request from the corresponding author.

## Supplementary Materials

The following supporting information can be downloaded at https://www.mdpi.com/article/10.3390/jcm14186436/s1: Figure S1: Summary of eLSS scores from computed tomography in study CLN01; Figure S2: Pediatric case 2 efficacy outcomes; Figure S3: Pediatric case 3 efficacy outcomes; Figure S4: Pediatric case 4 efficacy outcomes; Table S1: Treatment-emergent adverse events in study CLN01; Table S2: Treatment-emergent adverse events in pediatric cases treated with NVD003; Table S3: Individual eLSS scores from X-ray in study CLN01; Table S4: Mean and median total eLSS scores from X-ray in study CLN01.

## Abbreviations

The following abbreviations are used in this manuscript:

ASC	Adipose-derived mesenchymal stem cell
CPT	Congenital pseudarthrosis of the tibia
CSBD	Critical-sized bone defect
ECM	Extracellular matrix
ELISA	Enzyme-linked immunosorbent assay
eLSS	Extended Lane and Sandhu Scale
FDA	United States Food and Drug Administration
GMP	Good Manufacturing Practice
GS	Grafting surgery
HA/TCP	Hydroxyapatite/beta-tricalcium phosphate
IGF-1	Insulin-like growth factor-1
IHC	Immunohistochemistry
ODD	Orphan Drug Designation
rhBMP	Recombinant human bone morphogenic protein
μCT	Micro-computed tomography
VEGF	Vascular endothelial growth factor

## References

[1] Tsang S.J., Ferreira N., Simpson A. (2022). The reconstruction of critical bone loss: The holy grail of orthopaedics. Bone Joint Res..

[2] Nauth A., Schemitsch E., Norris B., Nollin Z., Watson J.T. (2018). Critical-size bone defects: Is there a consensus for diagnosis and treatment?. J. Orthop. Trauma..

[3] Fuchs T., Stolberg-Stolberg J., Michel P.A., Garcia P., Amler S., Wahnert D., Raschke M.J. (2021). Effect of bone morphogenetic Protein-2 in the treatment of long bone non-unions. J. Clin. Med..

[4] Feltri P., Solaro L., Di Martino A., Candrian C., Errani C., Filardo G. (2022). Union, complication, reintervention and failure rates of surgical techniques for large diaphyseal defects: A systematic review and meta-analysis. Sci. Rep..

[5] Andrzejowski P., Giannoudis P.V. (2019). The ‘diamond concept’ for long bone non-union management. J. Orthop. Traumatol..

[6] Ding Z.C., Lin Y.K., Gan Y.K., Tang T.T. (2018). Molecular pathogenesis of fracture nonunion. J. Orthop. Translat.

[7] Mouldavan F. (2019). Recent Trends in Bioprinting. Procedia Manuf..

[8] Xue C., Chen L., Wang N., Chen H., Xu W., Xi Z., Sun Q., Kang R., Xie L., Liu X. (2024). Stimuli-responsive hydrogels for bone tissue engineering. Biomater. Transl..

[9] Lin C.H., Srioudom J.R., Sun W., Xing M., Yan S., Yu L., Yang J. (2024). The use of hydrogel microspheres as cell and drug delivery carriers for bone, cartilage, and soft tissue regeneration. Biomater. Transl..

[10] RISystem RatFix Surgical Technique Guide. https://files.designer.hoststar.ch/3a/96/3a96539c-7eed-412c-8071-abb437d77137.pdf.

[11] Calori G.M., Colombo M., Mazza E.L., Mazzola S., Malagoli E., Marelli N., Corradi A. (2014). Validation of the non-union scoring system in 300 long bone non-unions. Injury.

[12] Masquelet A., Kanakaris N.K., Obert L., Stafford P., Giannoudis P.V. (2019). Bone repair using the masquelet technique. J. Bone Joint Surg. Am..

[13] Lane J.M., Sandhu H.S. (1987). Current approaches to experimental bone grafting. Orthop. Clin. N. Am..

[14] Fleiss J.L. (1971). Measuring nominal scale agreement among many raters. Psychol. Bull..

[15] Wittauer M., Burch M.A., McNally M., Vandendriessche T., Clauss M., Della Rocca G.J., Giannoudis P.V., Metsemakers W.J., Morgenstern M. (2021). Definition of long-bone nonunion: A scoping review of prospective clinical trials to evaluate current practice. Injury.

[16] Van Royen K., Brems H., Legius E., Lammens J., Laumen A. (2016). Prevalence of neurofibromatosis type 1 in congenital pseudarthrosis of the tibia. Eur. J. Pediatr..

[17] Zhu G., Zheng Y., Liu Y., Yan A., Hu Z., Yang Y., Xiang S., Li L., Chen W., Peng Y. (2019). Identification and characterization of NF1 and non-NF1 congenital pseudarthrosis of the tibia based on germline NF1 variants: Genetic and clinical analysis of 75 patients. Orphanet J. Rare Dis..

[18] Shah H., Rousset M., Canavese F. (2012). Congenital pseudarthrosis of the tibia: Management and complications. Indian. J. Orthop..

[19] Paley D. (2019). Congenital pseudarthrosis of the tibia: Biological and biomechanical considerations to achieve union and prevent refracture. J. Child. Orthop..

[20] Rastogi A., Agarwal A. (2022). Surgical treatment options for congenital pseudarthrosis of tibia in children: Cross-union versus other options: A systematic review. J. Pediatr. Orthop. B.

[21] Shannon C.E., Huser A.J., Paley D. (2021). Cross-union surgery for congenital pseudarthrosis of the tibia. Children.

[22] Zhang Y., Fan M., Zhang Y. (2024). Revolutionizing bone defect healing: The power of mesenchymal stem cells as seeds. Front. Bioeng. Biotechnol..

[23] Kim J.M., Lin C., Stavre Z., Greenblatt M.B., Shim J.H. (2020). Osteoblast-osteoclast communication and bone homeostasis. Cells.

[24] Reible B., Schmidmaier G., Moghaddam A., Westhauser F. (2018). Insulin-like growth factor-1 as a possible alternative to bone morphogenetic protein-7 to induce osteogenic differentiation of human mesenchymal stem cells in vitro. Int. J. Mol. Sci..

[25] Xian L., Wu X., Pang L., Lou M., Rosen C.J., Qiu T., Crane J., Frassica F., Zhang L., Rodriguez J.P. (2012). Matrix IGF-1 maintains bone mass by activation of mTOR in mesenchymal stem cells. Nat. Med..

[26] Hu K., Olsen B.R. (2016). The roles of vascular endothelial growth factor in bone repair and regeneration. Bone.

[27] De Pieri A., Rochev Y., Zeugolis D.I. (2021). Scaffold-free cell-based tissue engineering therapies: Advances, shortfalls and forecast. NPJ Regen. Med..

[28] Giannoudis P.V., Papakostidis C., Roberts C. (2006). A review of the management of open fractures of the tibia and femur. J. Bone Joint Surg. Br..

[29] Flierl M.A., Smith W.R., Mauffrey C., Irgit K., Williams A.E., Ross E., Peacher G., Hak D.J., Stahel P.F. (2013). Outcomes and complication rates of different bone grafting modalities in long bone fracture nonunions: A retrospective cohort study in 182 patients. J. Orthop. Surg. Res..

[30] Yang J., Zhang X., Liang W., Chen G., Ma Y., Zhou Y., Fen R., Jiang K. (2022). Efficacy of adjuvant treatment for fracture nonunion/delayed union: A network meta-analysis of randomized controlled trials. BMC Musculoskelet. Disord..

