# Supplementary material for: An Autologous Human Adipose Stem Cell-Derived 3D Osteogenic Implant for Bone Grafting: From Development to First-in-Human Experience
Source: J Clin Med. 2025 Sep 12;14(18):6436. doi: 10.3390/jcm14186436 (PMC12470636; doi:10.3390/jcm14186436)

Supplemental Materials

An Autologous Human Adipose Stem Cell-Derived 3D Osteogenic Implant for Bone Grafting: From Development to First-in-Human Experience

Supplemental Table S1 Treatment-emergent adverse events in Study CLN01

ID	Date of GS surgery	AE	SAE	Date of onset	Severity	Status	Relatedness to NVD003	Relatedness to surgery
PALU101	16/01/2019	Anemia	No	20/09/2019	Mild	Recovered /Resolved	Unrelated	Unrelated
		Urinary tract infection	No	20/09/2019	Mild	Recovered /Resolved	Unrelated	Unrelated
		Major bronchial secretion	No	20/09/2019	Mild	Recovered /Resolved	Unrelated	Unrelated
PALU102	04/07/2019	Left disc herniation L5-S1	No	28/12/2019	Severe	Recovered /Resolved	Unrelated	Unrelated
		Aggravated left disc herniation L5-S1	Yes	04/02/2020	Severe	Recovered /Resolved	Unrelated	Unrelated
PALU103	15/04/2020	Post-operative anemia	No	20/04/2020	Mild	Recovered /Resolved	Unrelated	Definitely related
		Post-operative pain	No	15/04/2020	Mild	Recovered /Resolved	Unrelated	Definitely related
		Hypotension	No	08/04/2021	Mild	Recovered /Resolved	Unrelated	Unrelated
PABE102	12/12/2018	Anemia post-surgery	No	13/12/2018	Severe	Recovered /Resolved	Unrelated	Probably related
		Increase of GOT	No	14/12/2018	Mild	Recovered /Resolved	Unrelated	Possibly related
		Increase CRP	No	13/12/2018	Mild	Recovered /Resolved	Unrelated	Definitely related
		Hypotension	No	15/12/2018	Mild	Recovered /Resolved	Unrelated	Probably related
		Edema limb left	No	22/01/2019	Mild	Recovered /Resolved	Unrelated	Possibly related
		Increase of GPT	No	14/12/2018	Mild	Recovered /Resolved	Unrelated	Possibly related
		Increase of GGT	No	14/12/2018	Mild	Recovered /Resolved	Unrelated	Possibly related
		Wounds slightly inflammatory	No	22/01/2019	Mild	Recovered /Resolved	Unrelated	Possibly related
		Diffuse left thigh pain	No	02/03/2020	Mild	Recovered /Resolved	Unrelated	Unlikely related
		Psoriasis on the palm of right hand	No	15/09/2020	Mild	Not recovered /Not resolved	Unrelated	Unrelated

ID	Date of GS surgery	AE	SAE	Date of onset	Severity	Status	Relatedness to NVD003	Relatedness to surgery
		Ductal carcinoma in situ	Yes	25/05/2023	Severe	Not Recovered / Not Resolved	Unrelated	Unrelated
		Breast reconstruction surgery	Yes	15/01/2024	Mild	Not recovered /Not resolved	Unrelated	Unrelated
PABE301	07/01/2019	Cold	No	11/01/2019	Mild	Recovered /Resolved	Unrelated	Unrelated
PABE501	15/02/2019	Edema of right leg (post-surgery)	No	21/02/2019	Mild	Recovered /Resolved	Unrelated	Probably related
PABE504	20/12/2019	Pain post-grafting surgery	No	20/12/2019	Mild	Recovered /Resolved	Unrelated	Possibly related
		Nausea (post-surgery)	No	20/12/2019	Mild	Recovered /Resolved	Unrelated	Possibly related
		Anterolateral left leg discomfort	No	21/01/2020	Mild	Not recovered /Not resolved	Unrelated	Unrelated
		Muscle pain anterolateral left leg	No	16/04/2020	Mild	Recovered /Resolved	Unrelated	Unrelated
		Radiologically observed NVD-003 graft remodeling, expected to be part of normal bone healing process at the level of external cortext of the middfle third of the left tibia	No	03/12/2020	Mild	Recovered /Resolved	Unlikely related	Unlikely related
		Increased pain at proximal level of the tibia	No	01/12/2020	Moderate	Recovered /Resolved	Unrelated	Unrelated
		Further deterioration of the	No	01/12/2020	Moderate	Recovered /Resolved	Unrelated	Unrelated

ID	Date of GS surgery	AE	SAE	Date of onset	Severity	Status	Relatedness to NVD003	Relatedness to surgery
		walking ability						
		Hyperten-sion	No	01/12/2020	Mild	Recovered /Resolved	Unrelated	Unrelated
PABE401	27/02/2020	Surgical wound erythema	No	11/03/2020	Mild	Recovered /Resolved	Unrelated	Definitely related
		Intermittent right ankle pain	Yes	23/03/2020	Severe	Recovered /Resolved	Unlikely related	Possibly related
		Refracture of the target tibial nonunion region	Yes	24/08/2021	Severe	Recovered /Resolved	Unrelated	Unlikely related
		Neuropathic pain	No	21/11/2021	Moderate	Not recovered /Not resolved	Unrelated	Unrelated
		Resection of a left breast tumor	Yes	24/02/2023	Severe	Recoverin g/Resolvin g	Unrelated	Unrelated
		Cicatricial disunion of the surgical wound on the right ankle	Yes	26/09/2023	Severe	Recovered /Resolved	Unrelated	Unlikely related
		Infectious colitis	Yes	02/03/2024	Severe	Recovered /Resolved	Unrelated	Unrelated
		Leukemia chronic myelo-monocytic	Yes	04/03/2024	Severe	Not recovered /Not resolved	Unrelated	Unrelated
PABE402	08/05/2020	Alanine amino-transferase increase	No	08/05/2020	Mild	Recovered /Resolved	Unrelated	Unrelated
		Aspartate amino-transferase increased	No	08/05/2020	Moderate	Recovered /Resolved	Unrelated	Unrelated
		Lactate dehydrog-enase increase	No	08/05/2020	Moderate	Recovered /Resolved	Unrelated	Unrelated
		Site surgery infection	No	02/06/2020	Mild	Recovered /Resolved	Unrelated	Unrelated

ID	Date of GS surgery	AE	SAE	Date of onset	Severity	Status	Relatedness to NVD003	Relatedness to surgery
		Worsening of staphylo-coccus aureus infection at surgical site	Yes	15/06/2020	Severe	Recovered /Resolved	Unrelated	Unrelated
		Costal fractures	No	01/10/2020	Moderate	Recovered /Resolved	Unrelated	Unrelated
		Gamma GT increasing	No	26/04/2021	Moderate	Not recovered /Not resolved	Unrelated	Unrelated
		Inflammatory pain	No	02/06/2020	Moderate	Recovered /Resolved	Unrelated	Unrelated
		Accumulation of subcutaneous fluid at the level of a screw in arthrodesis of right ankle	Yes	19/10/2021	Severe	Recovered /Resolved	Unrelated	Unrelated
		Deterioration of general status due to increase of alcohol consumption	Yes	06/08/2021	Severe	Recovered /Resolved	Unrelated	Unrelated
		Rhabdo-myolysis due to repeated fall	Yes	04/04/2022	Severe	Recovered /Resolved	Unrelated	Unrelated
		Surgical neck fracture of the right humerus following a fall	Yes	14/08/2023	Severe	Recovered /Resolved	Unrelated	Unrelated
		Heart attack	Yes	03/10/2023	Severe	Fatal	Unrelated	Unrelated

AE, adverse event; ID, participant identification; SAE: serious adverse event.

Supplemental Table S2 Adverse events in pediatric patients (Compassionate Use)

Patient	AE description	(S)AE seriousness	(S)AE intensity (Mild/ Moderate/ Severe)	(S)AE action taken	(S)AE outcome (recovered/ resolved or ongoing)	(S)AE relationship to study drug
**Case 1**	Pain in knee due to prominent nail	Yes	Mild	Hospitalization and corrective surgery to cut the most superior tibial intramedullary nail	Recovered/ Resolved	Unrelated to NVD003
**Case 1**	Pain in the ankle and bursitis at medial malleolus	Yes	Mild	Hospitalization and corrective surgery to cut the most inferior tibial intramodular nail	Recovered/ Resolved	Unrelated to NVD003
**Case 1**	Pain at the lateral malleolus	Yes	Mild	Hospitalization and corrective surgery to cut the fibular intramedullary nail	Recovered/ Resolved	Unrelated to NVD003
**Case 1**	Pain at the lateral malleolus	Yes	Mild	Hospitalization and corrective surgery to remove the fibular nail	Recovered/ Resolved	Unrelated to NVD003
**Case 2**	Left clavicle fracture	No	Mild	Splintage of upper arm	Recovered	Unrelated to NVD003
**Case 2**	Weakness (general status alteration)	Yes	Moderate	Sleep analysis (03-Feb-2020 and 04-Feb-2020). Brain and thoracic MRI on 19-Mar-2020.	Recovered/ Resolved	Unrelated to NVD003
**Case 2**	Endoscopic resection of a laryngeal neurofibroma	Yes	Moderate	None	Recovered/ Resolved	Unrelated to NVD003
**Case 2**	Wire migration	Yes	Moderate	Revision surgery (05-Feb-2021) (Fassier-Duval nailing + graft and proximal tibia osteotomy)	Recovered/ Resolved	Unrelated to NVD003
**Case 3**	Distal migration of the fibular nail	Yes	Moderate	Revision surgery to exchange the Fassier-Duval nail by a larger one	Recovered/ Resolved	Unrelated to NVD003
**Case 4**	Distal migration of the tibial nail and breakage of one of the 2 locking screws	Yes	Moderate	New surgery 29-Mar-2022: Removal of the broken locking screw	Recovered/ Resolved	Unrelated to NVD003

Supplemental Figure S1 Summary of eLSS scores from CT in Study CLN01

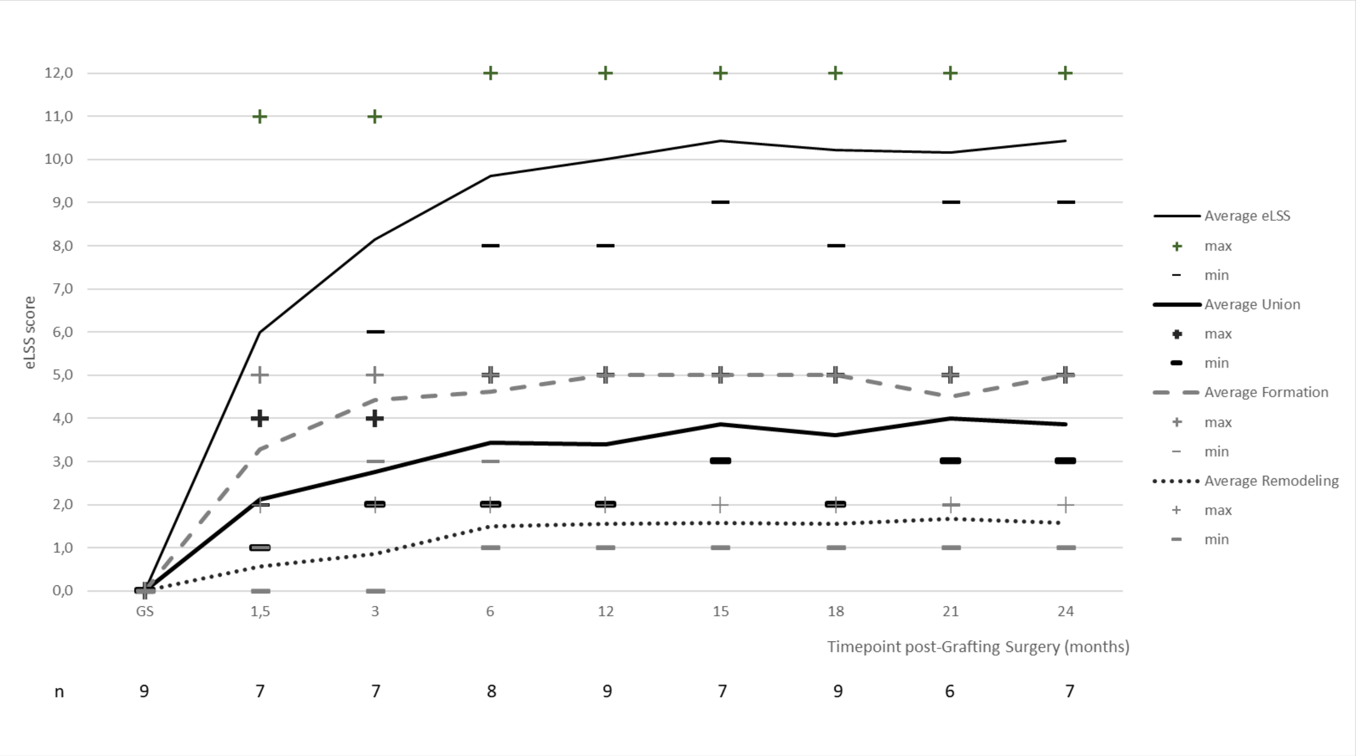

Extended Lane and Sandhu Scale (eLSS) scores for CT images following grafting surgery. The total eLSS score is the sum of three individual components: bone formation (longitudinal filling, 0-5), bone union (transverse filling, 0-5), and bone remodeling (continuity of bone architecture, 0-2).

Supplemental Table S3 Individual eLSS scores (X-ray)

	PABE102			PABE301			PABE401			PABE402			PABE501			PABE504			PALU101			PALU102			PALU103		
**Subscore**	Union	Formation	Remodelling	Union	Formation	Remodelling	Union	Formation	Remodelling	Union	Formation	Remodelling	Union	Formation	Remodelling	Union	Formation	Remodelling	Union	Formation	Remodelling	Union	Formation	Remodelling	Union	Formation	Remodelling
Screening	0	0	0	0	0	0	0	0	0	0	0	0	0	0	0	0	0	0	0	0	0	0	0	0	0	0	0
GS	0	0	0	0	0	0	0	0	0	NA	NA	NA	0	0	0	0	0	0	0	0	0	0	0	0	0	0	0
1.5	1	1	0	3	3	0	NA	NA	NA	NA	NA	NA	2	4	0	1	4	1	2	3	1	2	3	0	4	5	2
3	2	3	1	4	4	1	NA	NA	NA	NA	NA	NA	2	5	0	2	5	1	4	4	1	2	5	0	4	5	2
6	3	4	1	5	5	2	NA	NA	NA	5	3	1	3	5	1	2	5	2	3	5	2	3	5	1	NA	NA	NA
12	4	5	2	5	5	2	3	5	1	3	5	1	3	5	2	3	5	1	3	5	2	2	5	1	5	5	2
15	4	5	2	5	5	2	3	5	1	NA	NA	NA	ND	ND	ND	3	5	1	4	5	2	3	5	1	5	5	2
18	4	5	2	5	5	2	3	5	1	4	5	1	3	5	2	2	5	1	4	5	2	3	5	1	OU	OU	OU
21	3	5	2	OU	OU	OU	*2*	*4*	*1*	5	5	2	ND	ND	ND	NA	NA	NA	3	5	1	OU	OU	OU	OU	OU	OU
24	3	5	2	5	5	2	*3*	*5*	*1*	4	5	2	ND	ND	ND	3	5	1	4	5	1	3	5	1	5	5	2

Italic text indicates imaging was performed after NVD003 removal.
NA, images not available or not assessable (due to image quality and/or radiopacity); ND, visit not completed (protocol deviation); OU, visit not completed because union reported at previous visit (per protocol).

Supplemental Table S4 Mean and median total eLSS scores (X-ray)

	Mean (standard deviation)	Median (range)	Evaluable participants (N)
GS	0 (0)	0 (0)	8
1.5	6 (2.64)	6 (2-11)	7
3	8.1 (1.68)	8 (6-11)	7
6	9.6 (1.30)	9 (8-12)	8
12	10 (1.41)	10 (8-12)	9
15	10.4 (1.40)	11 (9-12)	7
18	10.2 (1.39)	10 (8-12)	9
21	10.2 (1.47)	9.5 (9-12)	6
24	10.4 (1.27)	10 (9-12)	7

Supplemental Figure S2 Pediatric Case 2 Efficacy Outcomes

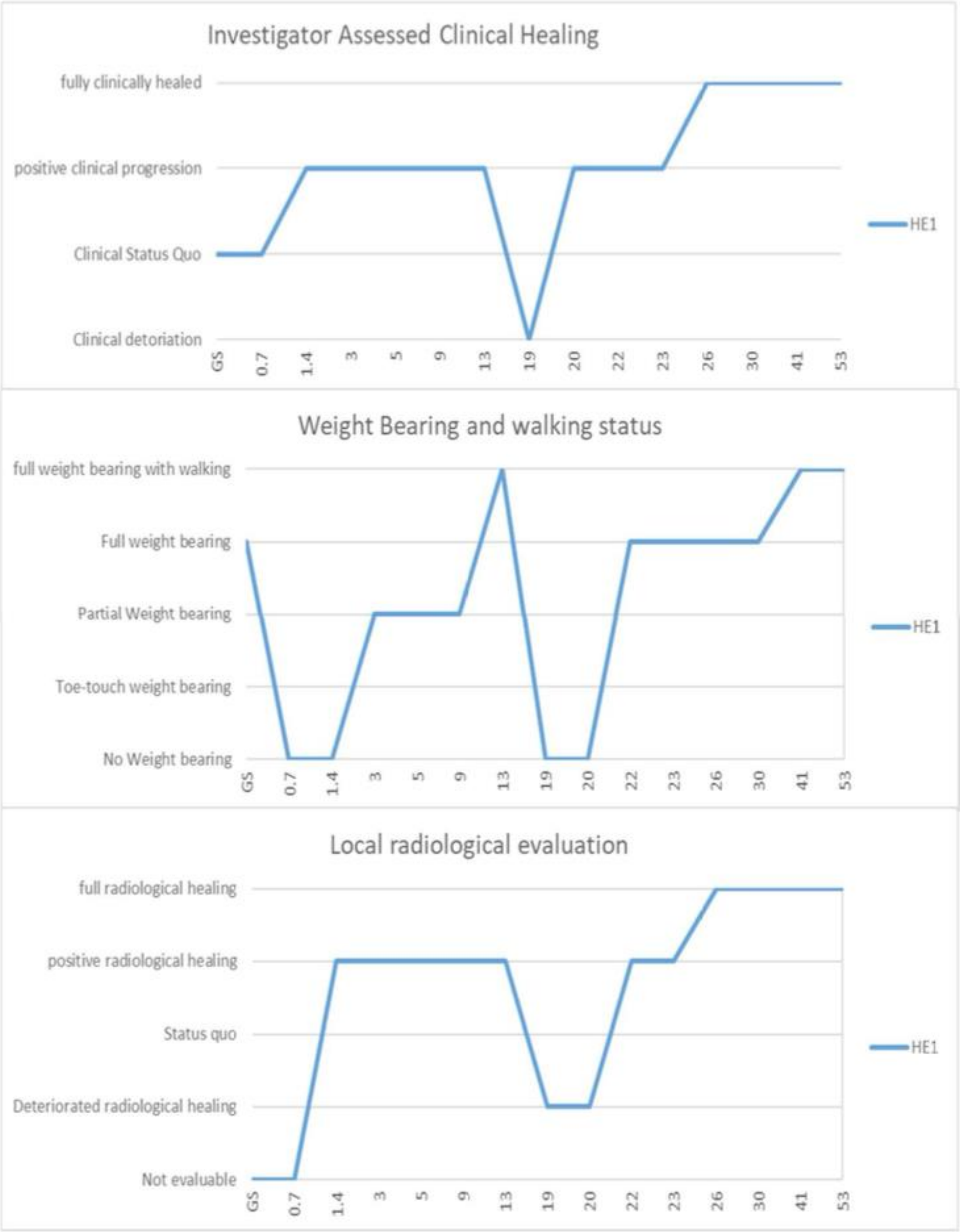

Supplemental Figure S3 Pediatric Case 3 Efficacy Outcomes

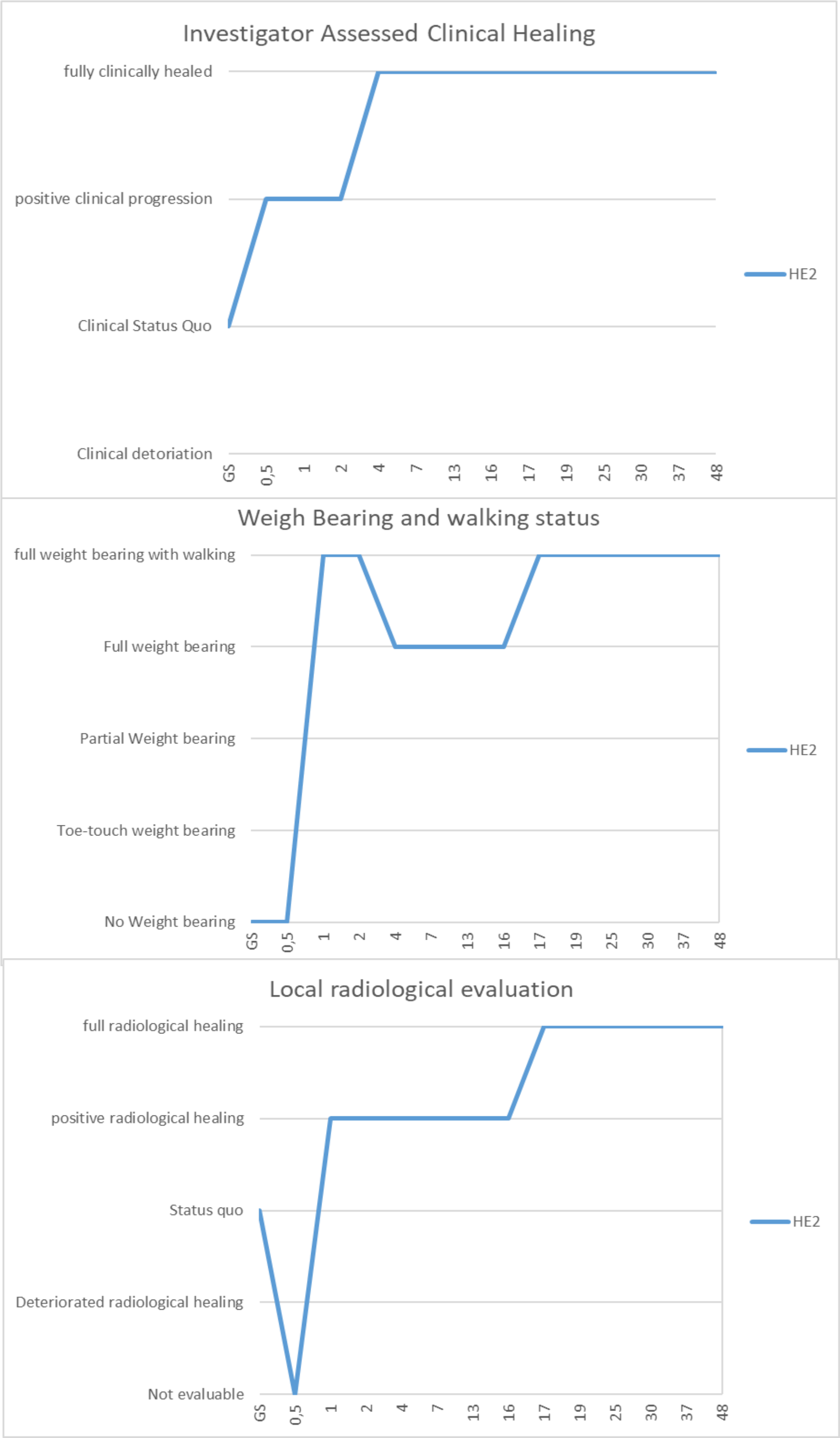

Supplemental Figure S4 Pediatric Case 4 Efficacy Outcomes

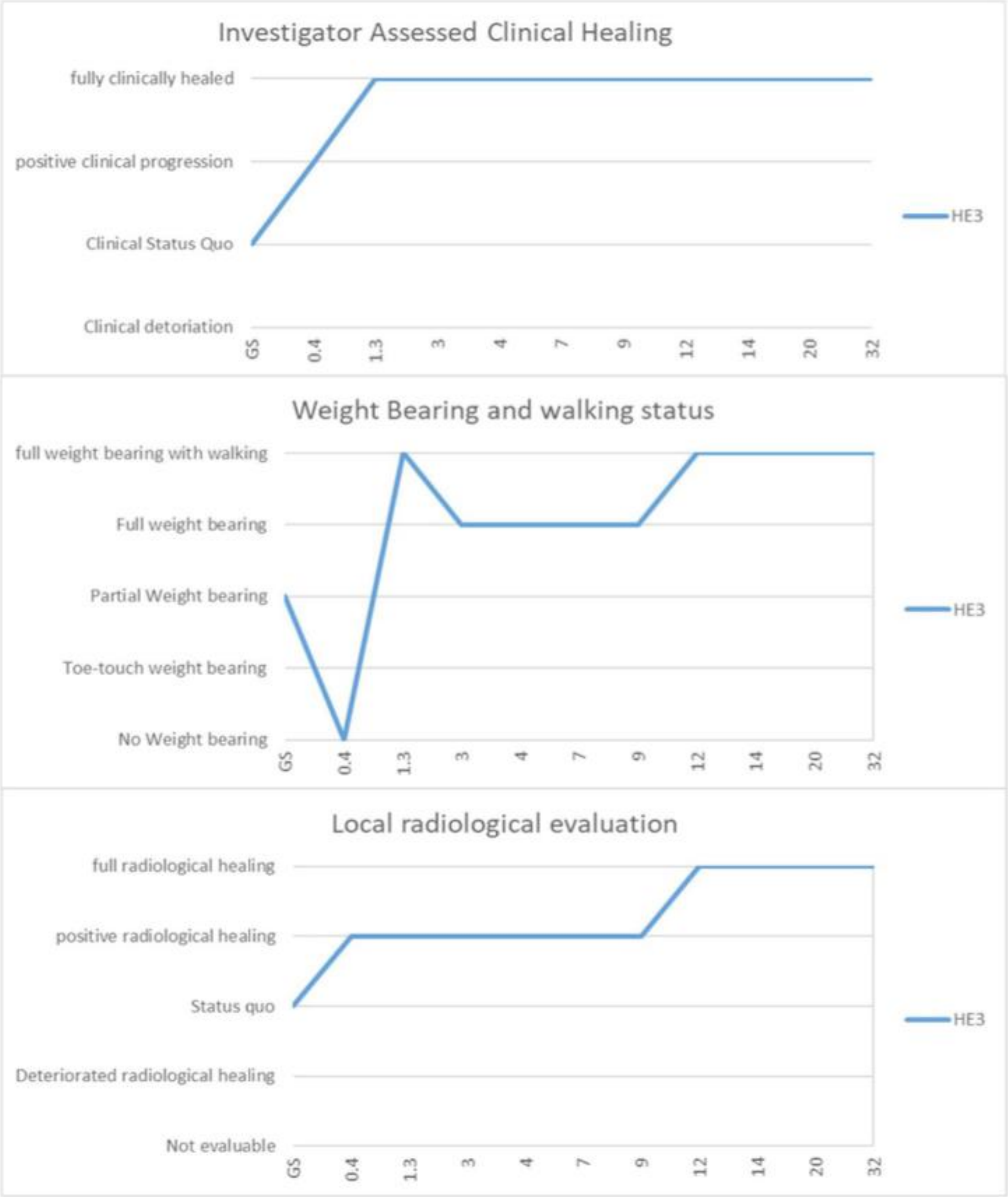

Supplement: Supplementary file 1 [file jcm-14-06436-s001.zip › jcm-3790383-supplementary.pdf]
